# RNA Size and Structure Modulate the Apparent p*K*_a_ of Ionizable Lipid Nanoparticles

**DOI:** 10.1021/acs.bioconjchem.6c00177

**Published:** 2026-07-29

**Authors:** Anastasiia Priss, Olha Lytvynenko, Vaclav Vanek, Frantisek Sedlak, Klara Grantz Saskova, Petr Cigler

**Affiliations:** † 89220 Institute of Organic Chemistry and Biochemistry of the Czech Academy of Sciences, Flemingovo nam. 2, 166 10 Prague, Czechia; ‡ Adalid Sciences, Podbabska 30, 160 00 Prague, Czechia

## Abstract

Ionizable lipid nanoparticles (LNPs) underpin today’s RNA medicines by shielding nucleic acids in the bloodstream and transforming into membrane-active cationic nanoparticles inside acidifying endosomes. This behavior critically depends on the apparent p*K*_a_ of LNPs, which can be tuned by altering the chemistry of lipids/lipidoids and the ratios of helper lipids. However, formulators typically assume that different RNA payloads exert identical effects on LNP ionization. In this study, we challenge this assumption and quantify how RNA modulates LNP p*K*_a_. LNP formulations with different ionizable lipidoids (SM-102, cKK-E12, ALC-0315, 306O_i10_, XMAN6, and C12-200) were used to encapsulate various RNA cargos, which ranged from 21-nt to 4.5-kb mRNAs. Apparent p*K*_a_ values were determined using a TNS fluorescence titration assay. RNA payload size impacted the apparent p*K*_a_ of the LNPs by 0.1–0.5 pH units, the direction and magnitude of the change being dependent on the structure of the ionizable lipidoid. Moreover, same-length 21-nt siRNA and miRNA cargos produced a 0.15 pH-unit difference in XMAN6 LNPs, indicating that RNA structural features can modulate apparent p*K*_a_ independently of payload size. With the monoamine lipidoid SM-102, short RNAs lowered the apparent p*K*_a_ relative to mRNA-loaded particles, whereas the multiamine lipidoids cKK-E12 and XMAN6 exhibited a monotonic decrease in apparent p*K*_a_ as RNA length increased, plateauing at ∼1.9 kb. The most pronounced discrepancies between apparent p*K*_a_ values of lipidoid particles and corresponding LNPs (of ≤0.5 pH units) were observed with the triamine lipidoid XMAN6, pointing to a role of amino group multiplicity in the modulation of apparent p*K*_a_. These findings demonstrate that RNA payload size is an underappreciated lever for tuning LNP ionization behavior and that considering RNA payload size when conducting lipid/lipidoid library screens could enhance the development of future RNA-based therapeutics.

## Graphical abstract

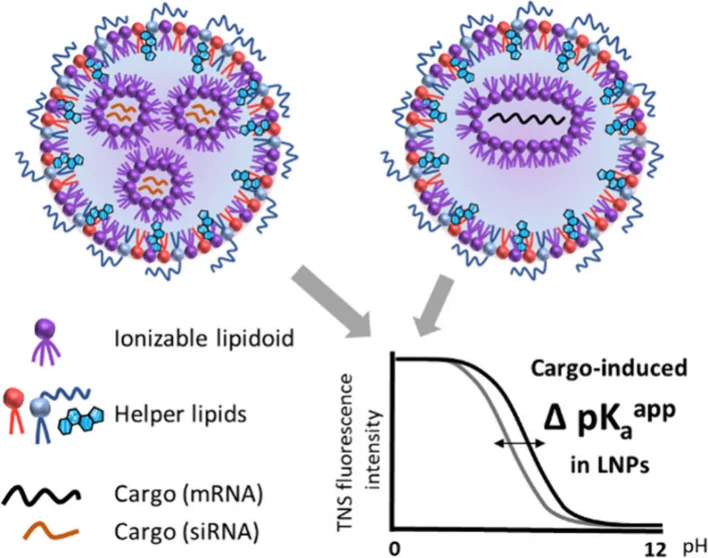

## Introduction

Recent advancements in RNA-based therapeutics encompass a wide variety of applications including vaccine development and protein replacement therapies. Gene silencing strategies allow for the controllable suppression of gene expression, whereas mRNA-based approaches enable transient protein expression, minimizing off-target effects, and long-term risks associated with permanent gene editing.^1–6^ Because RNA is susceptible to serum endonuclease degradation,^7^ efficient delivery systems are crucial for the development of therapeutics.^8^ To date, the highest transfection efficiency and lowest toxicity are provided by formulations referred to as lipid nanoparticle (LNP) RNA delivery systems, which have enabled the development of the antiamyloid drug Onpattro and of vaccines against the SARS-COV-2 virus.^9^

LNPs consist of ionizable lipids or lipidoids, phospholipids, cholesterol, and lipid-conjugated poly­(ethylene glycol) (PEG) (Figure 1A), forming spherical and multilamellar structures.^10^ Phospholipids and cholesterol support the encapsulation of RNA into lipid particles, stabilizing the complex. Additionally, phospholipids facilitate membrane fusion, whereas PEG–lipid conjugates affect the long-term stability of LNPs and minimize serum protein opsonization.^11–13^ Ionizable lipidoids become positively charged in acidic environments, such as inside endosomes, promoting endosomal escape and cytosolic delivery of encapsulated nucleic acids.^14^ Variations in the composition and proportions of lipids constituting LNPs significantly influence their tissue- and cell-targeting properties^15–17^ by directly influencing their charge and p*K*_a_.

**Figure 1:**
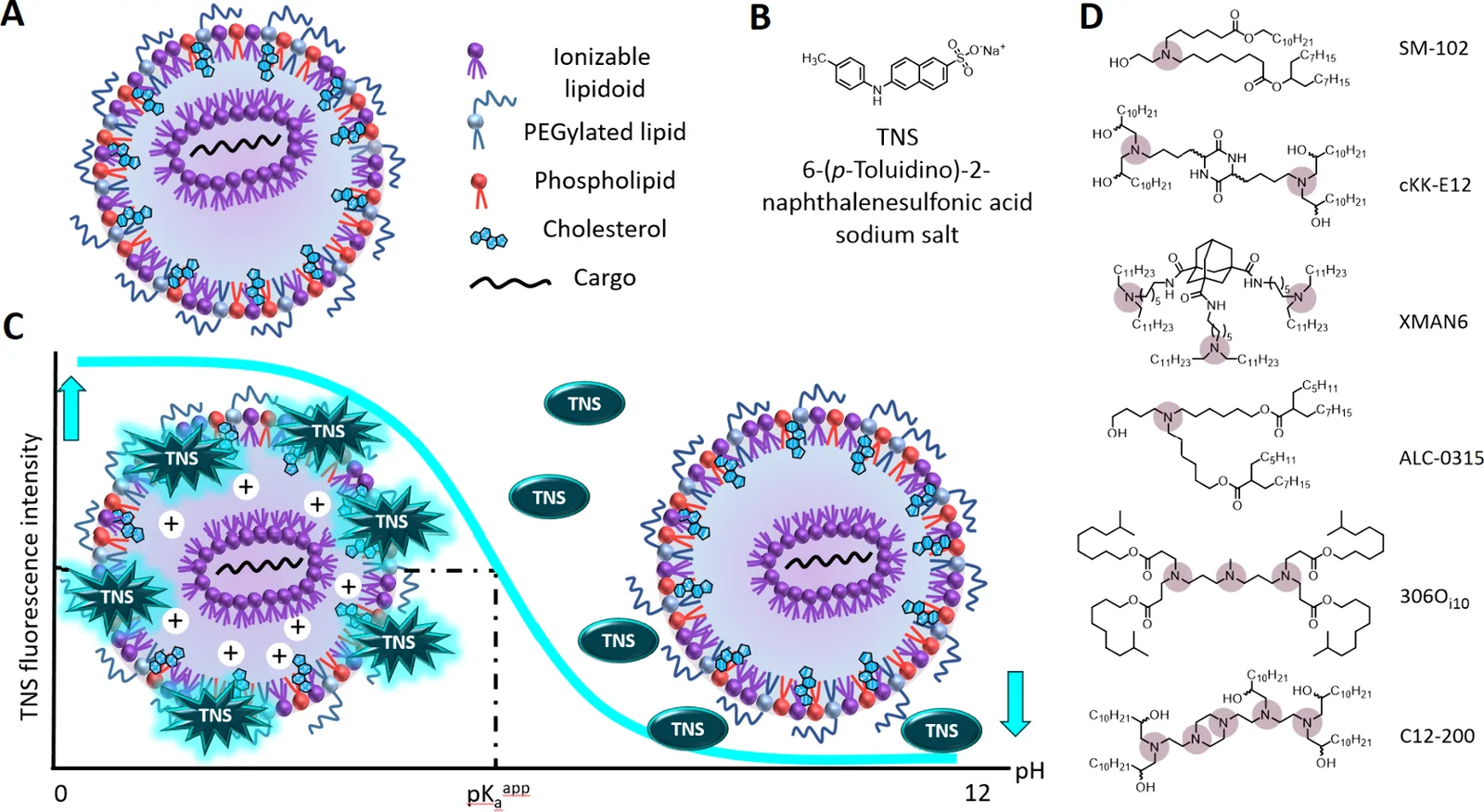
(A) Graphical representation of the composition of a lipid nanoparticle. (B) Chemical structure of the TNS fluorescent probe. (C) pH-dependence of the fluorescence response of the TNS probe upon its interaction with lipid nanoparticles. (D) Chemical structures of ionizable lipidoids used in the present study. Protonatable tertiary amino groups are highlighted.

The basicity of ionizable lipidoid headgroups strongly affects the ionization behavior of LNPs, influencing their stability, potency, and toxicity.^18^ The poor solubility of lipids in aqueous environments renders most methods for the accurate determination of their p*K*_a_ inapplicable, so indirect methods are utilized for studying the ionization behavior of their assemblies.^19^ One practical way to quantify the basicity/acidity of lipids in LNPs is to use the apparent dissociation constant p*K*_a_^app^ as determined by the 6-(*p*-toluidino)-2-naphthalenesulfonic acid (TNS) assay.^19^ This measure is widely adopted as a result of decades of studies of membrane surface potential and transport in phospholipid bilayers, as well as of the p*K*_a_ of liposomes.^20–22^

The p*K*_a_^app^ represents the pH at which half of the ionizable amino groups within the lipid nanoparticle are protonated, as inferred from the pH-dependent association of the anionic TNS dye with the nanoparticle surface. The TNS anionic sulfonate group associates electrostatically with positively charged lipids, whereas its hydrophobic part inserts into the lipid assembly, leading to increased fluorescence^19,22,23^ (Figure 1B).

Knowing the value of p*K*_a_^app^ is critical for optimizing endosomal processing and release. LNPs with siRNA cargo have been shown to exhibit a strong correlation between p*K*_a_^app^ and *in vivo* activity, with optimal potency observed in LNPs possessing a p*K*_a_^app^ between 6.2 and 6.5.^24^ Further toxicity studies have confirmed that LNPs with p*K*_a_^app^ values outside this optimal range can be excluded from *in vivo* testing, thereby conserving screening resources and reducing the use of animal models.^25^ Moreover, the degree of protonation at a late endosomal pH of approximately 5 has been identified as a critical factor for achieving high *in vivo* efficacy.^26^ The optimal p*K*_a_^app^ range was later found to differ between siRNA and mRNA therapeutics (with a slightly higher optimum of 6.6–6.9 for the latter),^27^ and efficient screening approaches based on p*K*_a_^app^ criteria are being developed rapidly.^28^

Endosomal pH differs significantly across cell types, and this variability leads to dramatic differences in LNP-mediated transfection outcomes. In a study on the targeting of LNPs by introducing additional lipids into the complex, lung-targeting LNPs exhibited much higher values of p*K*_a_^app^ (>9) than liver-targeting LNPs, whereas liver-targeting LNPs, conversely, had lower p*K*_a_^app^ (>6).^16^ Fine-tuning the p*K*_a_^app^ of LNPs offers a promising strategy to enhance mRNA delivery to cells of types that are otherwise difficult to transfect. This assumption is supported by data on differences in the targeting specificity of LNPs, the lipid composition of which affects the p*K*_a_^app^.^16,25^

The p*K*_a_^app^ of LNPs is commonly determined using fluorescence spectroscopy by measuring changes in fluorescence intensity as a function of pH. Typically, the fluorescence of TNS in the presence of LNPs is measured in series of buffers, providing a sigmoidal dependence. The value of p*K*_a_^app^ is then determined as the ‘midpoint’ pH (Figure 1C). Methods based on potentiometric titration,^19^ small-angle X-ray scattering (SAXS),^29^ or electrophoretic mobility^30^ have also been proposed in the literature; however, the TNS assay has gained widespread adoption due to its simplicity and the strong correlation between p*K*_a_^app^ values obtained by it and the *in vivo* activity of LNPs.

It is important to note that each p*K*_a_^app^ value determined by the TNS method describes the LNP system in its entirety and differs significantly from the calculated p*K*_a_ of neat ionizable lipids.^19,30,31^ Moreover, published p*K*_a_^app^ values for LNPs derived from the same ionizable lipid vary,^32^ which can be attributed to differences between experimental methodologies and to the pronounced effects of LNP composition and the process by which LNPs are assembled. Factors reported to influence p*K*_a_^app^ include nanoparticle curvature (which is related to size),^33^ PEG content,^34^ helper lipid ratios,^35^ and structural properties of ionizable lipids, such as the type of headgroup and tail.^36–38^ These variables collectively influence measured values of p*K*_a_^app^, highlighting the complexity of LNP characterization and the need for standardized approaches.

In the present article, we demonstrate that the size of the encapsulated RNA cargo affects the p*K*_a_^app^ of lipid nanoparticles, which, to the best of our knowledge, has not been reported before.

## Results and Discussion

RNA is the core therapeutic payload carried by LNPs, defining their purpose and driving the design and functionality of the lipid-carrier system. To investigate how different RNA cargos affect the p*K*_a_^app^ of LNPs, we tested three sets of LNPs based on the lipidoids SM-102, cKK-E12,^39^ and XMAN6 ^40^ (see Figure 1D for their chemical structures) loaded with different cargos, commonly used in LNP screening (TDP2 siRNA, poly­(A) RNA, and mKate2, FLuc, and Cas9 mRNA). The high encapsulation efficiency and uniform size distribution of the formulated LNPs (Tables S1 and S4 in the Supporting Information) demonstrate that their assembly was efficient at the given N/P ratio and that proper RNA charge neutralization took place. Altered encapsulation can therefore be ruled out as a factor that may influence the measured LNP p*K*_a_^app^.

The p*K*_a_^app^ values obtained for SM-102 mRNA particles (Figure 2A) correlate well with the commonly cited p*K*_a_^app^ value of 6.68 ^41^ (measured for LNPs with mRNA encoding influenza HA genes) and 6.6 ^27^ (measured for LNPs with FLuc mRNA). Encapsulation of siRNA and short poly­(A) strands, however, significantly decreases the p*K*_a_^app^ values of SM-102 LNPs.

**Figure 2:**
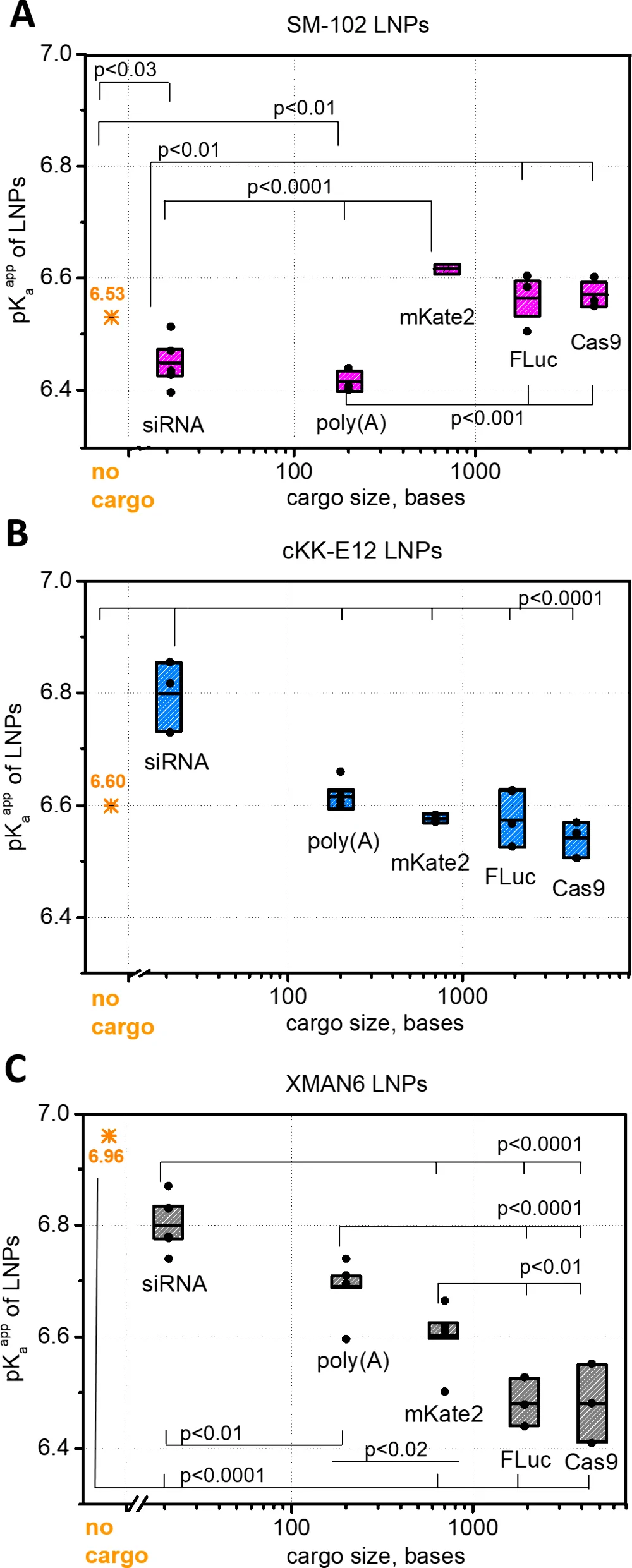
p*K*_a_^app^ of LNPs as a function of the size of RNA encapsulated in LNPs formulated with (A) SM-102, (B) cKK-E12, and (C) XMAN6. Each data point represents an independent formulation, horizontal lines indicate mean p*K*_a_^app^ values for each cargo group. The p*K*_a_^app^ of the corresponding ionizable lipidoid alone (i.e., without cargo; determined as described in ref 43) is indicated with an orange asterisk.

For cKK-E12 lipidoid nanoparticles formulated with FLuc-encoding mRNA, a p*K*_a_^app^ value of 6.51 ± 0.12 ^42^ has been reported, which corresponds to our observations (Figure 2B; Table S2 in the Supporting Information).

Depending on the ionizable lipidoid analyzed, our data show differences as large as 0.32 between mean p*K*_a_^app^ values of LNPs carrying different cargos (Figure 2). Moreover, for two of the lipidoids investigated, cKK-E12 and XMAN6, the detected shift in p*K*_a_^app^ correlated with the size of the cargo of LNPs (Figure 2B,C).

The p*K*_a_^app^ measured for LNPs carrying cKK-E12 and XMAN6 siRNA is 6.8, and this value drops noticeably with the increasing length of the cargo- siRNA (21 bp), poly­(A) (200 b) and mKate2 mRNA (740 b). In the case of XMAN6, an additional drop of 0.12 is observed between the p*K*_a_^app^ of mKate2 LNPs and FLuc (1929 b) LNPs. Interestingly, although the size of Cas9 mRNA is about 2.5 times greater than that of FLuc mRNA, no further change in LNP p*K*_a_^app^ was observed. Such a ‘plateau’ effect is supported by p*K*_a_^app^ values of hEpo- and Cre-mRNA-loaded XMAN6 LNPs (Table S2), which do not differ significantly from the p*K*_a_^app^ of FLuc LNPs.

Due to their large size, mRNA molecules carry a pronounced negative charge and can arrange into complex secondary and tertiary structures, which affect the spatial distribution of charge density. By contrast, poly­(A) RNA, being a homopolymer of adenine ribonucleotides, is incapable of intramolecular canonical base-pairing and therefore cannot form secondary structures. This limited ability to form secondary structures may be one of the reasons for the different ionization behaviors of these RNAs formulated in LNPs.

To assess the contribution of RNA secondary structure to the observed shifts in p*K*_a_^app^, LNPs containing either siRNA or miRNA (21 nt) were formulated with SM-102 and XMAN6 (Figure 3A). For SM-102 LNPs, no significant difference was observed between the two RNA types, whereas XMAN6-based LNPs exhibited a measurable difference of 0.15 pH units between siRNA- and miRNA-containing formulations. Furthermore, when comparing LNPs encapsulating mRNAs of similar length but different structure (mKate2, 740 nt; hEpo, 858 nt), the variation in p*K*_a_^app^ was again more pronounced for XMAN6 than for SM-102 (Figure 3A). These results indicate that, while differences in RNA secondary structure can induce measurable shifts in LNP p*K*_a_^app^ within a given lipid system, the magnitude of this effect strongly depends on the chemical nature of the ionizable lipidoid.

**Figure 3:**
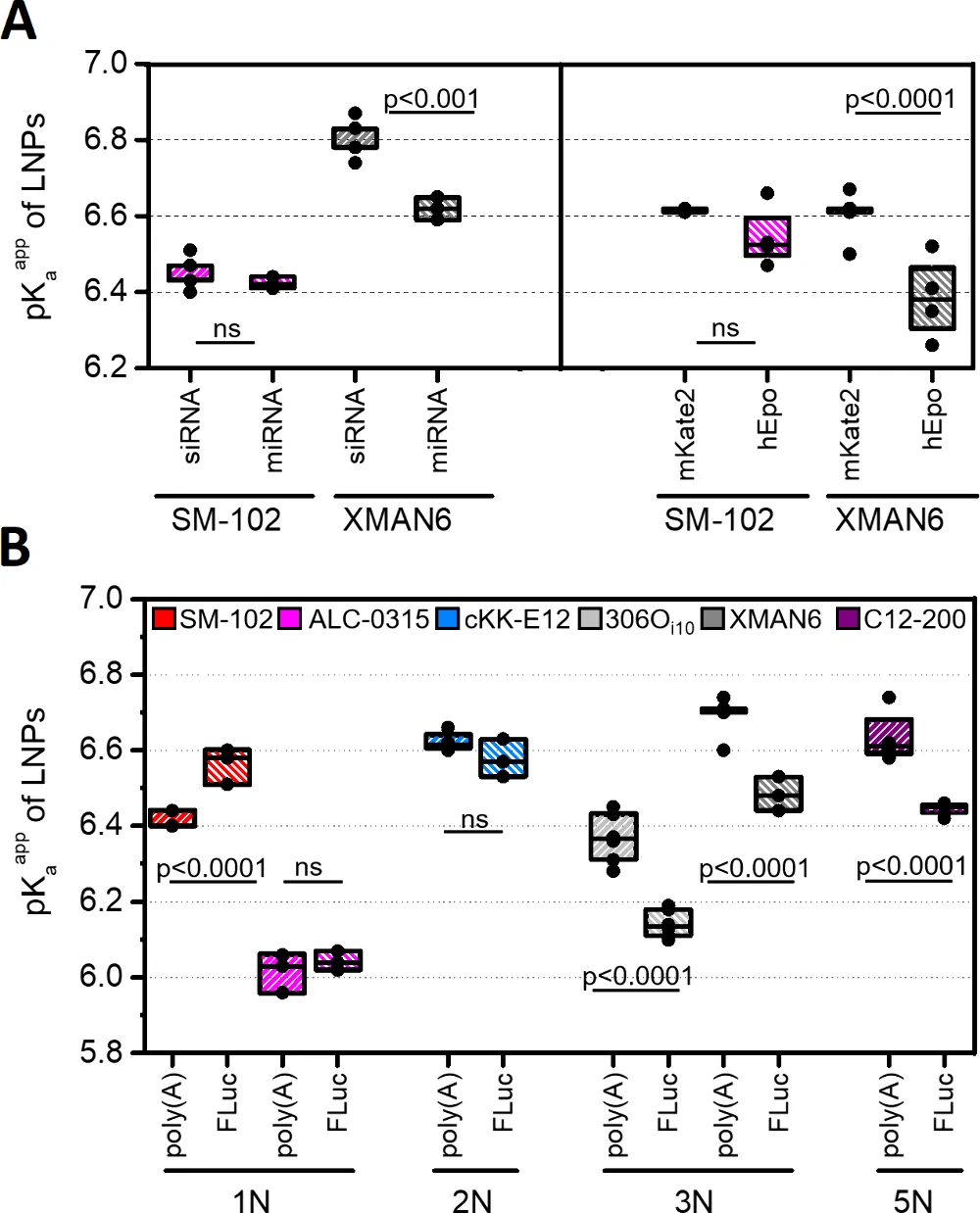
(A) p*K*_a_^app^ of SM-102 and XMAN6 LNPs formulated with different RNA payload. (B) p*K*_a_^app^ of LNPs, encapsulating poly­(A) and FLuc-encoding mRNA, formulated with monoamine and multiamine ionizable lipidoids. The number of ionizable amino groups (*N*) in each lipidoid is shown in the bottom. Each data point represents an independent formulation; horizontal lines indicate mean p*K*_a_^app^ values for each cargo group.

Helper lipid composition is known to affect the p*K*_a_^app^ of LNPs,^35^ with DOPE increasing lipid layer fluidity relative to DSPC. SM-102 LNPs are typically assembled with DSPC, whereas cKK-E12 and XMAN6 LNPs are assembled with DOPE; therefore we examined whether differences in ionization behavior could be influenced by this factor. SM-102 LNPs formulated with DOPE exhibited slightly lower p*K*_a_^app^ values for all tested RNAs compared with the corresponding SM-102/DSPC formulations (by 0.1–0.2 pH units). However, unlike in other DOPE-containing LNPs tested, no differences greater than 0.1 pH unit were observed between SM-102/DOPE LNPs formulated with different RNA cargos (Figure S5). Thus, although shifts in LNP p*K*_a_^app^ within the same lipid formulation can arise due to differences in the secondary structure of the loaded RNA, this effect depends strongly on the ionizable lipidoid used. Notably, the range and type of variation in LNP p*K*_a_^app^ that was caused by the RNA cargo also differ significantly between the ionizable lipidoids tested.

To increase the number of available platforms for commercial RNA delivery, ionizable lipids and lipidoids with diverse structures are being intensively investigated. Compounds with several ionizable amino groups have different protonation behaviors compared to those with one. LNPs derived from them often demonstrate enhanced RNA binding, endosomal escape, and delivery efficiency. In some cases, however, such LNPs are also more prone to being cytotoxic.^44^ Given the presence of two ionizable amino groups in the structure of cKK-E12 and three such groups in that of XMAN6, our data suggest that this structural feature might be behind the more prominent (compared to SM-102) dependence of LNP p*K*_a_^app^ on the cargo. This theory aligns well with a recent report that LNPs incorporating a triamine ionizable lipid displayed a stronger ionization response to variation in the lipid environment compared to LNPs formulated with monoamine lipids.^34^

To assess whether the p*K*_a_^app^ response to RNA cargo is conserved across other multiamine lipidoids, LNPs were formulated using 306O_i10_, bearing three ionizable amines,^45^ and C12-200, bearing five amines^46^ (Figure 1D). The p*K*_a_^app^ values obtained for FLuc-containing LNPs are in good agreement with literature reports for these lipidoids.^34,45^ Comparison of poly­(A)- and FLuc mRNA-loaded LNPs revealed cargo-dependent p*K*_a_^app^ shifts of ≥0.2 pH units in these additional multiamine systems (Figure 3B). We note that, for 306Oi10 LNPs, the TNS titration curves showed an additional feature in the acidic region below pH 4 (see Supporting Information, Figure S3); therefore, these low-pH points were omitted for all 306Oi10 data sets before fitting the principal sigmoidal transition. The absolute p*K*_a_^app^ values for 306Oi10 should consequently be interpreted with some caution. However, the same fitting procedure was applied to both RNA cargos, and the poly­(A)- versus FLuc-dependent shift was reproducible across independent formulations. Thus, the 306Oi10 data provide corroborating evidence for cargo-dependent modulation of p*K*_a_^app^ in multiamine LNPs. In contrast, LNPs formulated with the monoamine lipidoid ALC-0315 ^47^ did not exhibit a significant difference in p*K*_a_^app^ between the two RNA cargos. These findings support the conclusion that the sensitivity of LNP p*K*_a_^app^ to RNA-dependent effects increases with the number of ionizable amines in the lipidoid structure.

In addition to the p*K*_a_^app^ values, analysis of the Boltzmann fits revealed also systematic changes in the slope parameter ΔpH, which characterizes the width of the protonation transition. While ΔpH remained nearly constant for monoamine lipidoids, multiamine lipidoids showed an increase of ΔpH with increasing RNA cargo size, indicating a broader distribution of lipid protonation states in these systems. This effect likely reflects heterogeneous electrostatic interactions between ionizable lipid headgroups and the structurally complex RNA cargo (Table S3, Figure S2).

For screening, researchers favor fast, simple, and scalable methods of p*K*_a_ determination, so they often focus on the p*K*_a_ of the ionizable lipid/lipidoid, assumed most critical. However, the p*K*_a_^app^ of LNPs is reported to be 2–3 pH units lower than that of the respective ionizable lipids, as provided by NMR-based p*K*_a_ measurements or calculations.^30,31^ Accurate p*K*_a_ determination requires a low concentration of the ionizable lipid to minimize charge interactions. However, LNPs contain these lipids in abundance, and their tight packing limits accessibility for interactions with TNS and affects the p*K*_a_^app^. Predictions of p*K*_a_^app^ therefore retain significant uncertainty even in models for monoamines that incorporate corrections for environmental effects.^48,49^ More accurate predictions can be achieved through structure–activity relationship analysis within libraries of similar compounds, but this approach requires extensive experimental data.^36^

Although prediction models are not yet robust enough to replace experimental p*K*_a_^app^ determination, screens for new compounds are usually based on estimated p*K*_a_^app^ values of (single-component) ionizable cationic lipid assemblies, assuming similar ionization behavior to packed LNPs.^43^ However, our data indicate that the p*K*_a_^app^ value of LNPs is influenced by the entire environment of the complex, including the cargo itself. Even with the well-studied SM-102 lipidoid, RNA cargo slightly shifted the p*K*_a_^app^ values of particles (Figure 2A), with siRNA causing their decrease and mRNA their increase. This observation highlights the necessity to optimize every delivery system for the specific therapeutic task in order to maximize the efficiency.

For multiamine lipidoids, the discrepancy between the p*K*_a_^app^ of lipidoids alone and that of LNPs was found to be more prominent than for SM-102. In the case of the diamine lipidoid cKK-E12, the p*K*_a_^app^ of siRNA LNPs increased by 0.2 pH units compared to that of the lipidoid alone, whereas the p*K*_a_^app^ of mRNA LNPs was decreased (Figure 2B). LNPs based on triamine XMAN6 across all cargos showed LNP p*K*_a_^app^ values that were significantly lower than the lipidoid-alone p*K*_a_^app^ value, with differences of 0.5 of a pH unit (Figure 2C).

With lipidoids bearing multiple ionizable amino groups, TNS-based p*K*_a_^app^ determination can underestimate protonation at lower pH as a result of steric crowding by TNS molecules at the lipid surface. Consequently, increases in fluorescence may not scale with the number of protonated amino groups, even though interactions between neighboring amino groups still shape the p*K*_a_^app^ of the lipidoid assembly.^18,19^ Values of p*K*_a_^app^ obtained for multiamine lipidoids should therefore be interpreted cautiously when predicting structure–activity relationships of LNPs, as discrepancies between the p*K*_a_^app^ value of a lipidoid alone and that of assembled LNPs can be larger than for lipidoids containing a single ionizable amino group.

Our experiments show that the p*K*_a_^app^ values of LNPs are markedly affected by the encapsulation of RNA cargo, a kinetically controlled assembly process. This effect likely arises from location-dependent protonation within the nanoparticle;^50^ lipids in the hydrophobic core experience a p*K*_a_^app^ downshift due to headgroup repulsion and limited solvation, whereas lipids associated with RNA are stabilized by electrostatic interactions with the polyanionic backbone. As a result, RNA size and structure determine local phosphate density and packing, thereby modulating the balance between RNA-bound and core-localized lipids.^51^

Accordingly, p*K*_a_^app^ should be viewed as a continuum reflecting internal heterogeneity, rather than a single discrete value. Lipid shape and conicity further influence packing and the local protonation environment; even subtle geometric changes can compensate for intrinsic p*K*_a_ differences.^52^ Upon RNA binding and charge neutralization, changes in lipid geometry and particle curvature^53^ may additionally contribute to shifts in p*K*_a_^app^. Together, these structural features and the resulting water and ion accessibility govern LNP pH responsiveness and ultimately biological performance.

The demonstrated influence of RNA cargo on the p*K*_a_^app^ of LNPs, together with previous reports, underscores the complex interplay among all of the components comprising LNPs. While this effect should be considered when defining criteria for screening for new promising compounds, it also provides a way to more precisely tune the LNP p*K*_a_^app^ through both ionizable lipid design and lipid composition. Understanding how RNA cargo modulates ionization behavior, and, consequently, RNA delivery efficiency, expands the potential for rational LNP customization, including tissue-specific targeting and the co-delivery of multiple cargos for CRISPR-based therapeutics.

## Conclusion

Formulating LNPs with RNA cargos of different sizes shifts their p*K*_a_^app^ by 0.1–0.5 pH units, as demonstrated for SM-102, cKK-E12, XMAN6, 306O_i10_, and C12-200 LNPs in this study. For LNPs derived from the multiamine lipidoids cKK-E12 and XMAN6, the magnitude of the p*K*_a_^app^ shift correlates with the size of the encapsulated RNA. The comparison of 21-nt siRNA and miRNA cargos in XMAN6 LNPs further shows that, in this lipidoid system, cargo-dependent p*K*_a_^app^ modulation is not governed by RNA length alone but also reflects structure-dependent features of the encapsulated RNA. The fact that RNA cargo affects the p*K*_a_^app^ of LNPs composed of structurally distinct ionizable lipidoids, which is demonstrated by our results, highlights the complex interplay of factors governing the ionization behavior of LNPs and expands the potential for fine-tuning LNP properties for specific therapeutic applications.

## Experimental Procedures

### Materials

SM-102 (9-heptadecanyl 8-{(2-hydroxyethyl)­[6-oxo-6-(undecyloxy)­hexyl]­amino}­octanoate), cholesterol, DSPC (1,2-distearoyl-*sn*-glycero-3-phosphocholine), DOPE (2-dioleoyl-*sn*-glycero-3-phosphoethanol-amine), DMG-PEG_2000_ (1,2-dimyristoyl-*rac*-glycero-3-methoxypoly­(ethylene glycol)-2000), and cKK-E12 (3,6-bis­[4-[bis­(2-hydroxydodecyl)­amino]­butyl]-2,5-piperazinedione) were obtained from Avanti Polar Lipids. 306O_i10_ (tetrakis­(8-methylnonyl) 3,3′,3″,3‴-(((methylazanediyl)­bis­(propane-3,1-diyl))­bis­(azanetriyl))­tetrapropionate) was obtained from MedChemExpress. ALC-0315 ([(4-hydroxybutyl)­azanediyl]­di­(hexane-6,1-diyl) bis­(2-hexyldecanoate)) and C12-200 (1,1′-((2-(4-(2-((2-(bis­(2-hydroxydodecyl)­amino)­ethyl)­(2-hydroxydodecyl)­amino)­ethyl)­piperazin-1-yl)­ethyl)­azanediyl)­bis­(dodecan-2-ol)) were obtained from BLDpharm. XMAN6 (*N* ^1^,*N* ^3^,*N* ^5^-tris­(6-(didodecylamino)­hexyl)­adamantane-1,3,5-tricarboxamide) was synthesized as described earlier.^40^ Tyrosyl-DNA Phosphodiesterase 2 (TDP2) siRNA and scrambled miRNA (ThermoFisher scientific) were used as 21-nt cargo; Cas9, Cre, and hEpo mRNAs were obtained from TriLink BioTechnologies, and poly­(A) was obtained from Cytiva; mKate2 and FLuc mRNA were expressed and produced by *in vitro* transcription, as described earlier.^40^

### LNP Formulation

Lipid nanoparticles were assembled by microfluidic mixing of ethanol phase, containing 5 mM lipid mixtures (total concentration), and aqueous solution of RNA. Mixing was done using a “Y” herringbone microfluidic device with two inputs and one output (at mixing rate of 300 μL/min). Formulation parameters were based on the lipidoid-specific recommendations from literature sources (Table 1). After assembly, the LNPs were immediately diluted 2-fold in PBS while mixing. No unexpected or unusually high safety hazards were encountered.

**Table 1:** Formulation Parameters for Poly­(A) and mRNA LNP Assemblies with Different Lipidoids

	Lipidoid, %	Cholesterol, %	DMG-PEG, %	helper lipid, %	mRNA buffer	N/P ratio
SM-102 ^54^	50	38.5	1.5	10 DSPC	10 mM acetate, pH 4	6
ALC-0315 ^47^	46.3	42.8	1.5	9.4 DSPC		6
cKK-E12 ^55^	50	38.5	1.5	10 DOPE		6
XMAN6 ^40^	22	43.5	1.5	33 DOPE	10 mM citrate, pH 3	3
306O_i10_ ^45^	35	47.5	1.5	16 DOPE		6
C12-200 ^46^	35	47.5	1.5	16 DOPE		15

For assembling LNPs with siRNA and miRNA, N/P 27 was used for all lipidoids (see Supporting Information 4, Figure S4). LNP quality was checked by assessing encapsulation efficiency using Quant-IT RiboGreen RNA assay kit and size was checked using a NanoZS Zetasizer, Malvern (Table S1 in the Supporting Information). Assemblies were performed in triplicate for miRNA, FLuc, and Cas9 LNPs and in higher replication for LNPs with other cargos (Table S1).

### Determination of the p*K*_a_^app^ of Ionizable Lipidoids

The p*K*_a_^app^ of lipidoids was measured as described previously.^43^ Briefly, buffer solutions with pH values of 4.5, 5.27, 6.19, 6.57, 7.15, 7.76, 8.3, and 10.5 were prepared using a buffer system consisting of 20 mM sodium phosphate, 25 mM sodium citrate, 25 mM sodium acetate, and 150 mM sodium chloride (the pH was adjusted using 6 M aqueous NaOH or HCl). A solution of the lipidoid (0.5 mM) and TNS (0.15 mM) in an ethanol–methanol mixture was added to the buffers in a 3:97 (v/v) ratio and dispensed into replicate wells of a well plate for further fluorescence readout (λ_ex_ = 325 nm, λ_em_ = 435 nm, Tecan Spark plate reader). The obtained fluorescence intensities were baseline-corrected and normalized to the lowest-pH plateau, plotted against pH values and fitted with a sigmoid function to calculate the p*K*_a_^app^ value (Table S6). The fitting was performed in OriginLab Software with the Boltzmann function:

$$I=\frac{I_{1}-I_{2}}{1+e^{(\mathrm{pH}-{pK}_{a}^{\mathrm{app}})/\Delta \mathrm{pH}}}+I_{2}$$

where *I*_1_ and *I*_2_ are the intensities of the two plateaus, ΔpH characterizes the steepness of the transition region between these plateaus, and p*K*_a_^app^ corresponds to the inflection point of the transition region.

### Determination of the p*K*_a_^app^ of LNPs

A buffer containing 10 mM HEPES, 10 mM MES, 10 mM ammonium acetate, and 130 mM sodium chloride was used to obtain a series of solutions with pH values in the range of 2.5–11.^24^ These buffers were dispensed into wells in tetraplicates (95 μL per well). Assembled LNPs (5 μL) and 5 μL of 60 μM TNS were then introduced to buffers using a Mantis liquid dispenser. After mixing and a total incubation time of 5 min, the TNS fluorescence intensity was measured, plotted, and fitted to calculate p*K*_a_^app^ values, as described above.

### Statistical Analysis

The statistical significance of differences between groups was determined by two-way ANOVA with the Tukey’s multiple comparison test.

## Supplementary Material



## Notes

(1) Characteristics of LNPs used for the p*K*_a_^app^ evaluation, including the encapsulation efficiency and size of LNPs; (2) LNP p*K*_a_^app^ measurement details, including a full list of determined p*K*_a_^app^ and ΔpH values; (3) commentary to the fitting of sigmoid to 306Oi10 LNP titration data; (4) commentary on the N/P ratio impact on p*K*_a_^app^ of LNPs; (5) p*K*_a_^app^ of LNPs formulated with SM-102/DOPE. (6) DLS characteristics of lipidoids/TNS assemblies (PDF)
