# Supplementary material for: RNA Size and Structure Modulate the Apparent pK a of Ionizable Lipid Nanoparticles
Source: Bioconjug Chem. 2026 Jul 29;37(8):1537–44. doi: 10.1021/acs.bioconjchem.6c00177 (PMC13495550; doi:10.1021/acs.bioconjchem.6c00177)
Supplement: Supplementary file 1 [file bc6c00177_si_001.pdf]

Supporting information

RNA Size and Structure Modulate the Apparent pK_a of Ionizable Lipid Nanoparticles

Anastasiia Priss[1,#], Olha Lytvynenko[1,#], Vaclav Vanek[1], Frantisek Sedlak[2], Klara Grantz Saskova[2], Petr Cigler[1,2,*]

[1] Institute of Organic Chemistry and Biochemistry of the CAS, Flemingovo nam. 2, 166 10 Prague, Czechia
[2] Adalid Sciences, Podbabska 30, 160 00 Prague, Czechia

[#] These authors contributed equally.
* Corresponding author, E-mail: cigler@uochb.cas.cz

1. Characterization of the lipid nanoparticles

Dynamic light scattering (DLS) measurements were performed using the Zetasizer Nano ZS (Malvern Panalytical) with a particle RI of 1.45 and an absorption value of 0.001 in PBS at 25 °C with a viscosity of 0.888 cP and and RI of 1.335. Measurements were conducted using disposable cuvettes, with a 173° backscatter detection angle. Each measurement consisted of 10 runs with a 10 s run duration, without delay between measurements. The particle size is represented as volume-weighted hydrodynamic diameter, D_h.

Encapsulation efficiency was assessed by Quant-it™ RiboGreen reagent (ThermoFischer). Intact LNPs were measured in Tris (10 mM, pH 7.5)/EDTA (1 mM) (TE) at a 1:99 volume ratio (premixed 2.5:247.5 µl and further added in duplicates as 100 µL per well on a black microplate). Free RNA was measured from LNPs, treated with Triton/TE (2% v/v Triton in TE buffer) at a 1:19 volume ratio (premixed 4:76 µl, incubated for 10 min at 37 °C, and added to the same microplate in duplicates as 20 µL per well. The volume was further adjusted to 100 µl by TE.
Two standard curves were included in the Quant-it™ RiboGreen assay, one containing mRNA in TE, and the other containing mRNA in Triton/TE. Each standard curve was used to calculate the mRNA concentration in each corresponding buffer. The Ribogreen reagent was diluted 1:100 in TE buffer and added to the wells at a 1:1 volume ratio. Fluorescence readout from the microplates was performed using a Tecan microplate reader (λ_{ex} 485 nm, λ_{em} 528 nm). RNA concentration was estimated based on the standard curve, and the encapsulation efficiency, EE(%), was calculated using the equation:

$$EE(\%) = (1 - [\text{Free RNA}]/[\text{RNA in intact LNPs}]) \times 100\%.$$

For miRNA, RNA concentration in samples with intact LNPs and LNPs, treated in Triton/TE was measured by Qubit miRNA Assay kit (ThermoFischer), according to manufacturer's protocol. Further calculations were done same as with RiboGreen assay.

Table S1. Characteristics of the assembled LNPs. The results of hydrodynamic diameter D_h and encapsulation efficacy EE are expressed as mean values over the number of assemblies (*N*) ± standard deviation. DLS data for lipidoid-alone assemblies are shown below in section 6.

Ionizable lipid	Cargo	Length (number of bases)	Number of assemblies *N*	D_h (nm)	Encapsulation efficiency EE (%)
SM-102	TDP2 siRNA	21	5	95 ± 8	92.4 ± 0.8
	miRNA	21	3	76 ± 3	89.6 ± 0.7
	poly(A)	~200	3	124 ± 8	96.4 ± 0.5
	mKate2 mRNA	740	3	109 ± 4	83.5 ± 13.0
	FLuc mRNA	1929	3	133 ± 26	95.9 ± 0.5
	Cas9 mRNA	4521	3	117 ± 9	95.3 ± 0.5
	hEpo mRNA	858	4	115 ± 19	83.5 ± 2.7
cKK-E12	TDP2 siRNA	21	3	110 ± 3	84.8 ± 4.3
	poly(A)	~200	4	125 ± 9	95.9 ± 0.6
	mKate2 mRNA	740	4	118 ± 1	85.0 ± 4.2
	FLuc mRNA	1929	3	126 ± 4	91.7 ± 0.6
	Cas9 mRNA	4521	3	131 ± 7	88.6 ± 1.1
XMAN6	TDP2 siRNA	21	5	96 ± 13	89.0 ± 3.7
	miRNA	21	3	79 ± 4	85.1 ± 2.9
	poly(A)	~200	5	120 ± 5	94.0 ± 4.2
	mKate2 mRNA	740	5	105 ± 12	92.3 ± 3.5
	FLuc mRNA	1929	3	118 ± 9	92.3 ± 3.5
	Cas9 mRNA	4521	3	112 ± 9	88.6 ± 8.2
	hEpo mRNA	858	4	120 ± 11	94.1 ± 1.8
	Cre mRNA	1350	1	100	97.2
ALC-0315	poly(A)	~200	3	89 ± 2	77.7 ± 0.1
	FLuc mRNA	1929	3	90 ± 2	84.2 ± 2.2
306Oi10	poly(A)	~200	6	142 ± 18	77.7 ± 5.9
	FLuc mRNA	1929	6	153 ± 15	80.9 ± 3.2
C12-200	poly(A)	~200	4	160 ± 15	77.6 ± 4.2
	FLuc mRNA	1929	4	128 ± 14	79.7 ± 0.7

2. LNP pK_a^{app} measurements

Experimental results were fitted as exemplified in Figure S1 and summarized in Table S2.

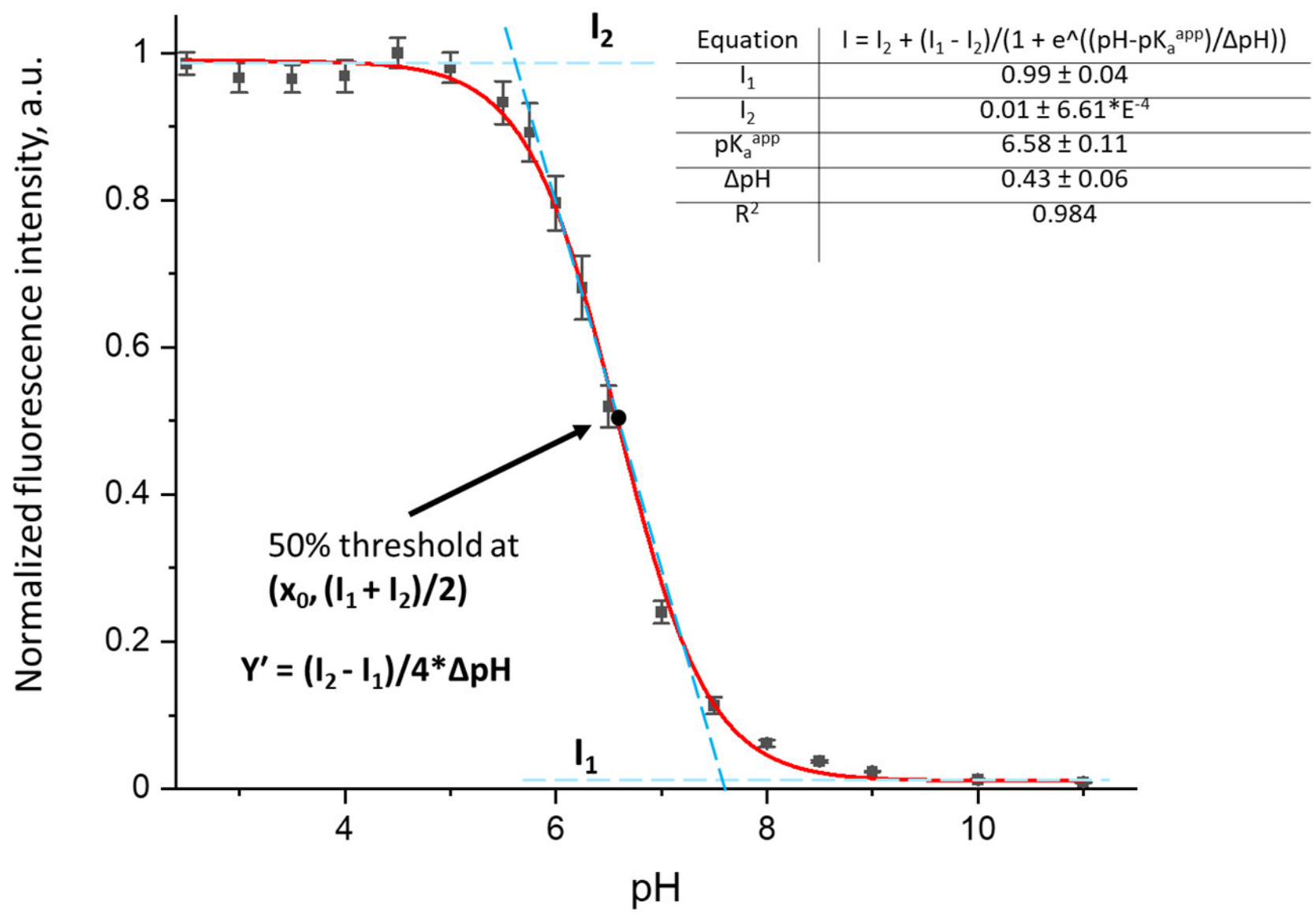

Figure S1. Representative curve analysis for pK_a^{app} determination (based on one of the replicates of cKK-E12 mKate2 LNP assemblies). Error bars on the graph represent SD, derived from technical replicates of each point (n = 4).

Table S2. pK_a^{app} of the assembled LNPs with different cargos.

LNP with	siRNA	poly(A)	mKate2	FLuc	Cas9	hEpo	miRNA
SM - 102	6.43 ± 0.03	6.41 ± 0.07	6.62 ± 0.05	6.60 ± 0.02	6.60 ± 0.02	6.52 ± 0.04	6.41 ± 0.05
	6.43 ± 0.03	6.44 ± 0.02	6.61 ± 0.04	6.58 ± 0.03	6.55 ± 0.04	6.47 ± 0.03	6.44 ± 0.05
	6.51 ± 0.03	6.40 ± 0.08	6.62 ± 0.09	6.51 ± 0.02	6.56 ± 0.05	6.53 ± 0.05	6.42 ± 0.05
	6.40 ± 0.02					6.66 ± 0.02	
	6.47 ± 0.05						
cKK-E12	6.73 ± 0.06	6.66 ± 0.14	6.58 ± 0.11	6.53 ± 0.06	6.51 ± 0.08		
	6.82 ± 0.04	6.60 ± 0.07	6.58 ± 0.09	6.57 ± 0.07	6.55 ± 0.06		
	6.85 ± 0.04	6.61 ± 0.11	6.57 ± 0.09	6.63 ± 0.05	6.57 ± 0.05		
		6.62 ± 0.04	6.58 ± 0.09				
XMAN6	6.78 ± 0.03	6.71 ± 0.03	6.62 ± 0.07	6.48 ± 0.04	6.48 ± 0.05	6.52 ± 0.04	6.62 ± 0.05
	6.74 ± 0.06	6.70 ± 0.04	6.50 ± 0.02	6.53 ± 0.06	6.55 ± 0.05	6.47 ± 0.03	6.59 ± 0.04
	6.78 ± 0.08	6.60 ± 0.07	6.67 ± 0.06	6.44 ± 0.03	6.41 ± 0.08	6.53 ± 0.05	6.65 ± 0.05
	6.83 ± 0.08	6.71 ± 0.06	6.61 ± 0.04	Cre: 6.47 ± 0.04		6.52 ± 0.03	
	6.87 ± 0.09	6.74 ± 0.03	6.62 ± 0.09				

LNP with	siRNA	poly(A)	mKate2	FLuc	Cas9	hEpo	miRNA
ALC-0315		6.06 ± 0.03		6.02 ± 0.08			
		6.03 ± 0.04		6.07 ± 0.04			
		5.96 ± 0.01		6.04 ± 0.04			
306O i10		6.28 ± 0.07		6.11 ± 0.06			
		6.31 ± 0.04		6.10 ± 0.10			
		6.43 ± 0.06		6.14 ± 0.04			
		6.45 ± 0.05		6.18 ± 0.12			
		6.36 ± 0.07		6.19 ± 0.05			
		6.37 ± 0.04		6.13 ± 0.07			
C12-200		6.66 ± 0.03		6.45 ± 0.03			
		6.74 ± 0.05		6.46 ± 0.04			
		6.58 ± 0.03		6.42 ± 0.03			
		6.62 ± 0.04		6.45 ± 0.10			

Table S3. ΔpH of the assembled LNPs with different cargos.

LNP with	siRNA	poly(A)	mKate2	FLuc	Cas9	hEpo	miRNA
SM - 102	0.37 ± 0.02	0.37 ± 0.02	0.38 ± 0.02	0.40 ± 0.01	0.39 ± 0.01	0.39 ± 0.02	0.32 ± 0.04
	0.39 ± 0.01	0.35 ± 0.01	0.38 ± 0.04	0.40 ± 0.01	0.40 ± 0.01	0.38 ± 0.01	0.31 ± 0.03
	0.34 ± 0.01	0.38 ± 0.02	0.37 ± 0.02	0.45 ± 0.01	0.48 ± 0.02	0.49 ± 0.02	0.34 ± 0.03
	0.35 ± 0.01					0.38 ± 0.03	
	0.38 ± 0.02						
cKK-E12	0.52 ± 0.02	0.52 ± 0.04	0.52 ± 0.03	0.48 ± 0.03	0.53 ± 0.03		
	0.47 ± 0.01	0.48 ± 0.03	0.53 ± 0.03	0.53 ± 0.02	0.56 ± 0.02		
	0.53 ± 0.02	0.51 ± 0.03	0.52 ± 0.03	0.48 ± 0.02	0.58 ± 0.02		
		0.46 ± 0.03	0.51 ± 0.03				
XMAN6	0.52 ± 0.01	0.55 ± 0.01	0.58 ± 0.03	0.65 ± 0.02	0.66 ± 0.02	0.66 ± 0.03	0.50 ± 0.03
	0.55 ± 0.03	0.57 ± 0.02	0.60 ± 0.02	0.66 ± 0.04	0.62 ± 0.02	0.64 ± 0.03	0.48 ± 0.04
	0.5 ± 0.03	0.55 ± 0.03	0.58 ± 0.03	0.63 ± 0.01	0.60 ± 0.02	0.68 ± 0.02	0.51 ± 0.03
	0.46 ± 0.04	0.53 ± 0.03	0.61 ± 0.02	Cre: 0.70 ± 0.03		0.60 ± 0.02	
	0.43 ± 0.05	0.53 ± 0.03	0.61 ± 0.02				

LNP with	siRNA	poly(A)	mKate2	FLuc	Cas9	hEpo	miRNA
ALC-0315		0.34 ± 0.03		0.41 ± 0.03			
		0.38 ± 0.03		0.41 ± 0.03			
		0.40 ± 0.01		0.44 ± 0.04			
306O i10		0.42 ± 0.04		0.57 ± 0.04			
		0.50 ± 0.02		0.58 ± 0.03			
		0.47 ± 0.05		0.54 ± 0.04			
		0.48 ± 0.03		0.56 ± 0.03			
		0.46 ± 0.04		0.58 ± 0.04			
		0.54 ± 0.04		0.60 ± 0.04			
C12-200		0.44 ± 0.03		0.55 ± 0.03			
		0.54 ± 0.02		0.53 ± 0.02			
		0.50 ± 0.04		0.56 ± 0.03			
		0.46 ± 0.03		0.54 ± 0.02			

Properties of ΔpH values

The parameter ΔpH describes the steepness of the protonation transition and is inversely proportional to the slope of the curve at pH = pK_a^{app}. For an ideal single protonation equilibrium described by the Henderson–Hasselbalch equation, the theoretical value of ΔpH is 1/ln 10 ≈ 0.434, which follows from the identity $10^x = e^{x \ln 10}$. In practice, values of ΔpH close to 0.43 correspond to nearly ideal Henderson–Hasselbalch behavior, whereas larger values (≈ 0.5–1) indicate increasing heterogeneity or a broader distribution of protonation states within the lipid assembly. Deviations from the theoretical value therefore reflect non-ideal protonation behavior arising, for example, from heterogeneity of lipid microenvironments, electrostatic interactions between neighboring ionizable groups, or the presence of multiple protonatable amines within the lipidoid headgroup.

The ΔpH values obtained from our fits are presented in Table S3 and depicted in Figure S2. The observed trend suggests an increase of ΔpH values with RNA size. This effect likely arises from heterogeneous electrostatic interactions between ionizable lipid headgroups and the structurally complex RNA molecule, where local variations in phosphate density and RNA secondary structure modulate lipid protonation equilibria. As a result, larger RNA cargos produce a wider range of effective lipid pK_a^{app} values and consequently a broader protonation transition. Notably, this behavior was observed for lipidoids containing multiple ionizable amines, whereas monoamine lipidoids did not show a comparable dependence on RNA size. This observation is consistent with sequential and partially coupled protonation of multiple amine groups in multiamine lipidoids, where electrostatic interactions with the polyanionic RNA and between neighboring protonated amines broaden the protonation transition. In contrast, monoamine lipidoids with a single ionizable site exhibit a more uniform ionization behavior.

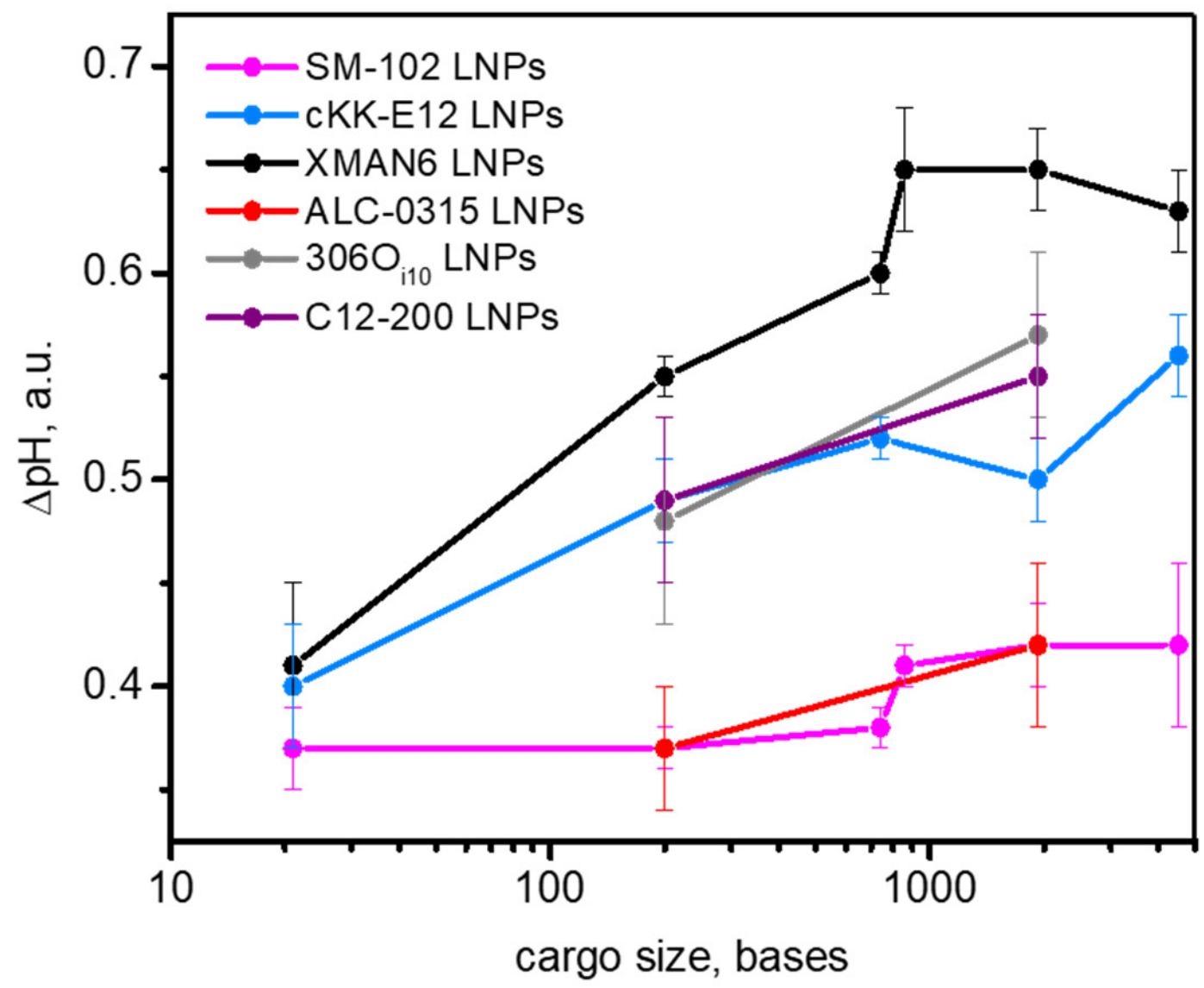

Figure S2. ΔpH of fitted titration curves as a function of the size of encapsulated LNPs, formulated with different lipidoids. Error bars on the graph represent SD of values across each group of samples.

3. Determination of pK_a^{app} for $306O_{i10}$ LNPs

Lipid nanoparticles based on $306O_{i10}$ lipidoid (three ionizable amino groups) exhibited unusual behavior in the acidic region in the buffer system used (Figure S3). We assume it could be caused by the protonation of the least basic amine in the molecule. To obtain a more accurate sigmoidal fit for the pK_a^{app} determination, data points below pH 4 were omitted for all samples containing this lipidoid (both poly(A) and FLuc-encoding mRNA). A comparison of the two fitting curves is presented in Figure S3. For the data reported in the paper, the “Boltzmann fit 1” approach was used.

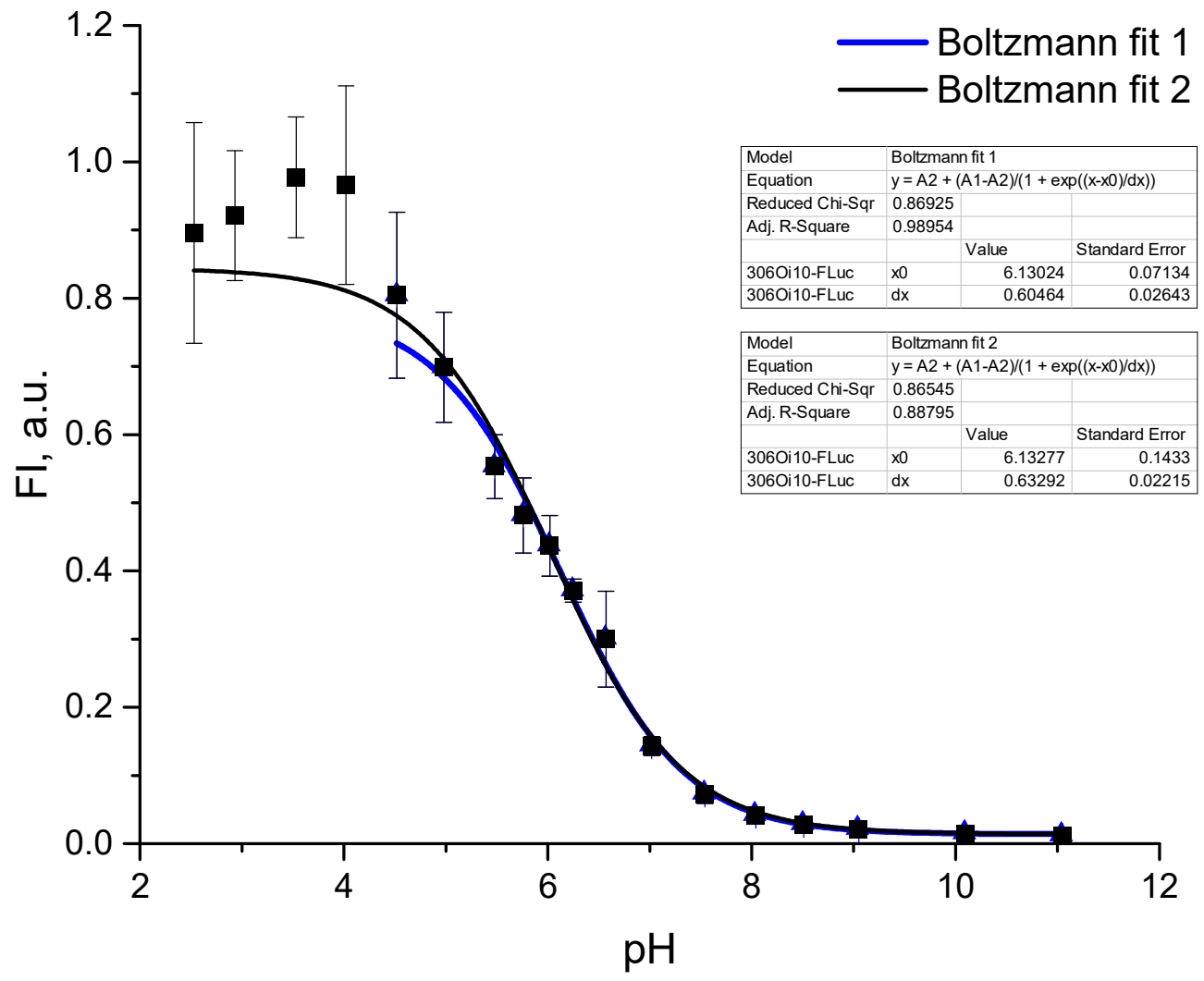

Figure S3. Representative curve analysis for pK_a^{app} determination of $306O_{i10}$-based LNPs. Error bars on the graph represent standard deviation derived from technical replicates of each point (n = 4).

4. Influence of N/P ratio on pK_a^{app} of LNPs.

Depending on the lipid system, efficient N/P ratios for mRNA encapsulation vary (3 to 15, and even more), therefore in this study we used the N/P ratios recommended for each compound (see Experimental section). Complexation of siRNA, however, differs from complexation of mRNA, favoring significantly higher N/P ratios for multiamine lipidoids. As an N/P ratio of 3 to 6 does not provide high-quality siRNA LNPs for all tested lipidoids, the N/P ratio for siRNA (and miRNA) was fixed across all groups to a commonly used low-payload formulation with N/P = 27. Here, we tested whether the reported payload effect on pK_a^{app} is linked to the N/P difference between formulated siRNA and mRNA LNPs. One of the multiamine lipidoids with a pronounced pK_a^{app} difference between siRNA and mRNA LNPs (XMAN6) was used to encapsulate FLuc mRNA as described previously, but with an N/P ratio of 27, and the pK_a^{app} of these LNPs was compared with those of the standard formulations. The obtained data demonstrated no significant impact of the N/P ratio change on the pK_a^{app} of LNPs with the same payload.

Table S4. Characteristics and pK_a^{app} of the XMAN6 - FLuc LNPs with N/P=27.

Ionizable lipid	N/P ratio	Cargo	Length (number of bases)	Number of assemblies *N*	D_h (nm)	Encapsulation efficiency EE (%)	pK_a^{app}
XMAN6	27	FLuc mRNA	1929	3	86 ± 8	61.1 ± 3.2	6.39 ± 0.04
							6.52 ± 0.06
							6.44 ± 0.05

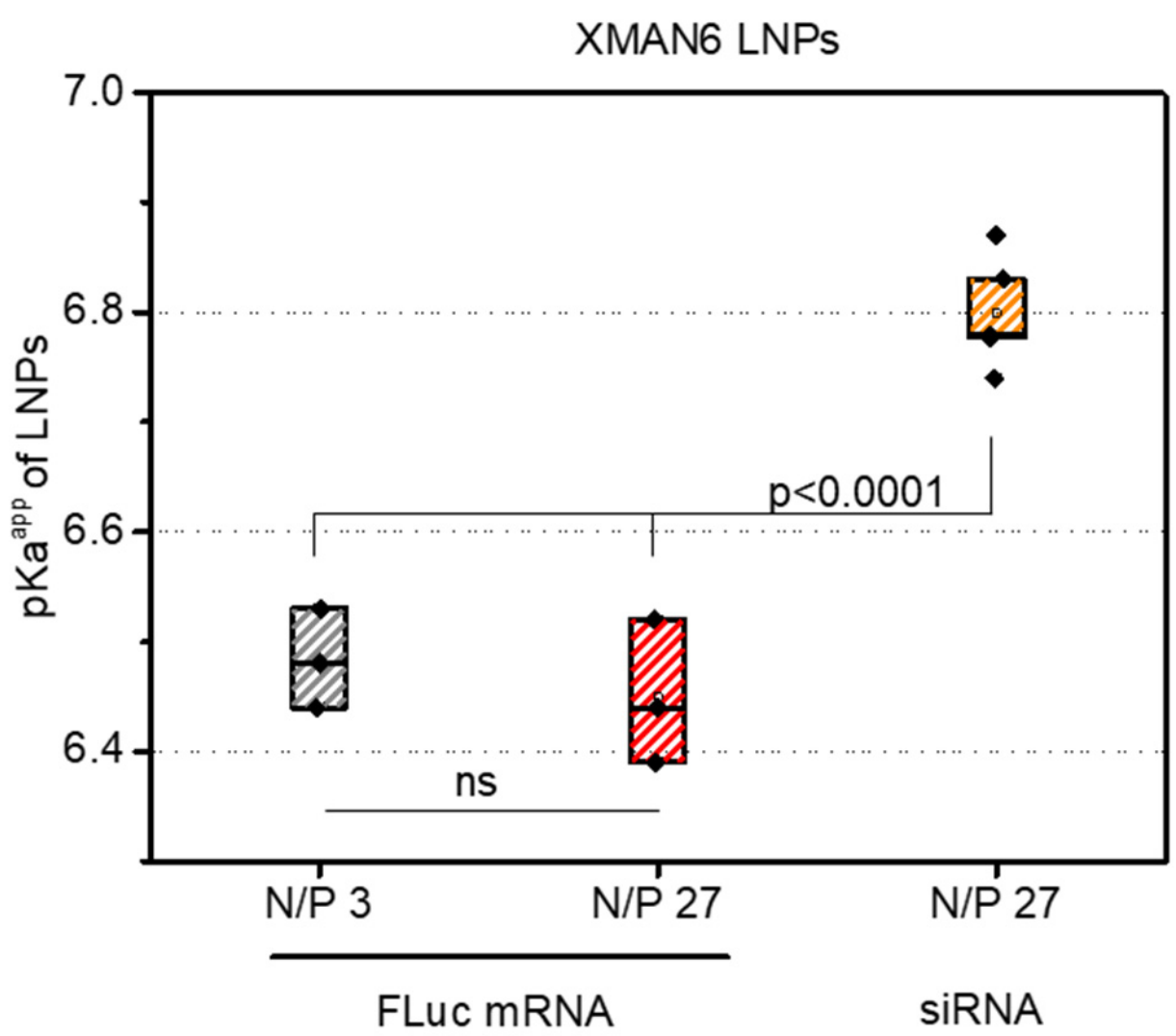

Figure S4. pK_a^{app} of LNPs formulated with FLuc mRNA at different N/P ratios and siRNA LNPs. Each data point represents an independent formulation.

5. Influence of DOPE helper lipid on the pK_a^{app} of LNPs with different payload.

Formulation of SM-102/DOPE LNPs was performed in the same way as the classical formulation with DSPC. LNPs were assembled using microfluidic mixing of an aqueous phase containing RNA and an ethanol phase containing 5mM lipid mixture. The following molar ratios were used to obtain the lipid mixture: SM-102:cholesterol:DOPE:DMG-PEG2000 = 50:38.5:10:1.5.

Table S5. Characteristics of the LNPs with SM-102 ionizable lipid assembled with DOPE helper lipid. Hydrodynamic diameter D_h, encapsulation efficacy EE, and pK_a^{app} of the particles are presented as mean values over the number of assemblies (*N*) ± standard deviation.

Formu-lation	Cargo	D_h (nm)	EE (%)	pK_a^{app}		
SM-102/DOPE	TDP2 siRNA	79 ± 3	79.9 ± 0.9	6.39 ± 0.06	6.26 ± 0.06	6.29 ± 0.07
	poly(A)	124 ± 9	85.3 ± 1.2	6.46 ± 0.03	6.37 ± 0.08	6.47 ± 0.07
	mKate2 mRNA	117 ± 6	80.7 ± 0.8	6.36 ± 0.03	6.35 ± 0.02	6.36 ± 0.03
	FLuc mRNA	171 ± 16	71.0 ± 2.8	6.45 ± 0.03	6.48 ± 0.03	6.46 ± 0.02
	Cas9 mRNA	185 ± 10	73.4 ± 2.0	6.36 ± 0.05	6.34 ± 0.06	6.48 ± 0.03

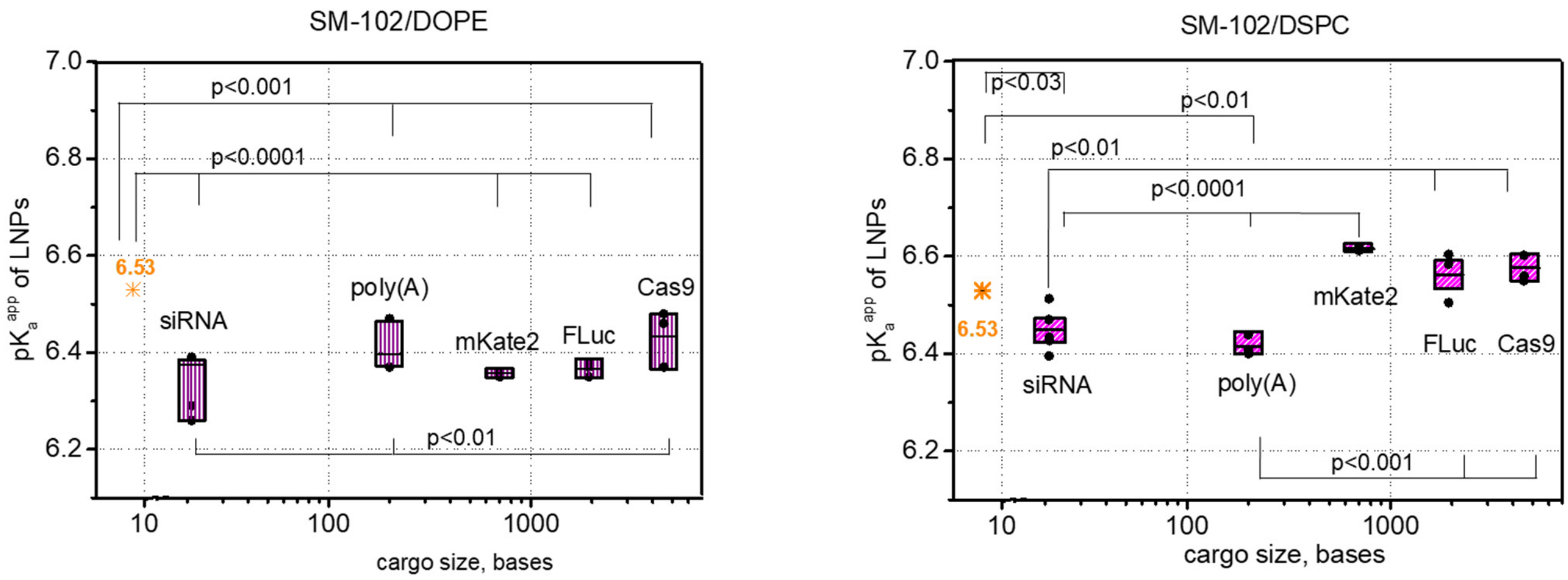

Figure S5. pK_a^{app} of LNPs with SM-102 ionizable lipidoid formulated with DOPE and DSPC.

6. Characteristics of ionizable lipidoid/TNS assemblies

Table S6. pK_a^{app} values determined for ionizable lipidoids only (without cargo and helper lipids). Mean values over the number of assemblies (*N*) ± standard deviation are presented. The pK_a^{app} of the ionizable lipidoids was determined as previously described[1].

Ionizable lipid	pK_a^{app}
ALC-0315	6.08 ± 0.08
SM-102	6.53 ± 0.08
cKK-E12	6.60 ± 0.10
306Oi10	5.88 ± 0.07
XMAN6	6.96 ± 0.09
C12-200	6.87 ± 0.08

The DLS measurement of self-assemblies of the ionizable lipid after association with TNS was performed at two selected pH (5.3 and 10.5). Stock buffer containing 20 mM sodium phosphate, 25 mM sodium citrate, 25 mM sodium acetate, and 150 mM sodium chloride, was adjusted to pH 5.3 or 10.5 with HCl or NaOH solutions, respectively. The size of lipidoid-only assemblies after interaction with TNS was monitored by dynamic light scattering using a Zetasizer Nano ZS (Malvern Panalytical), as described above.

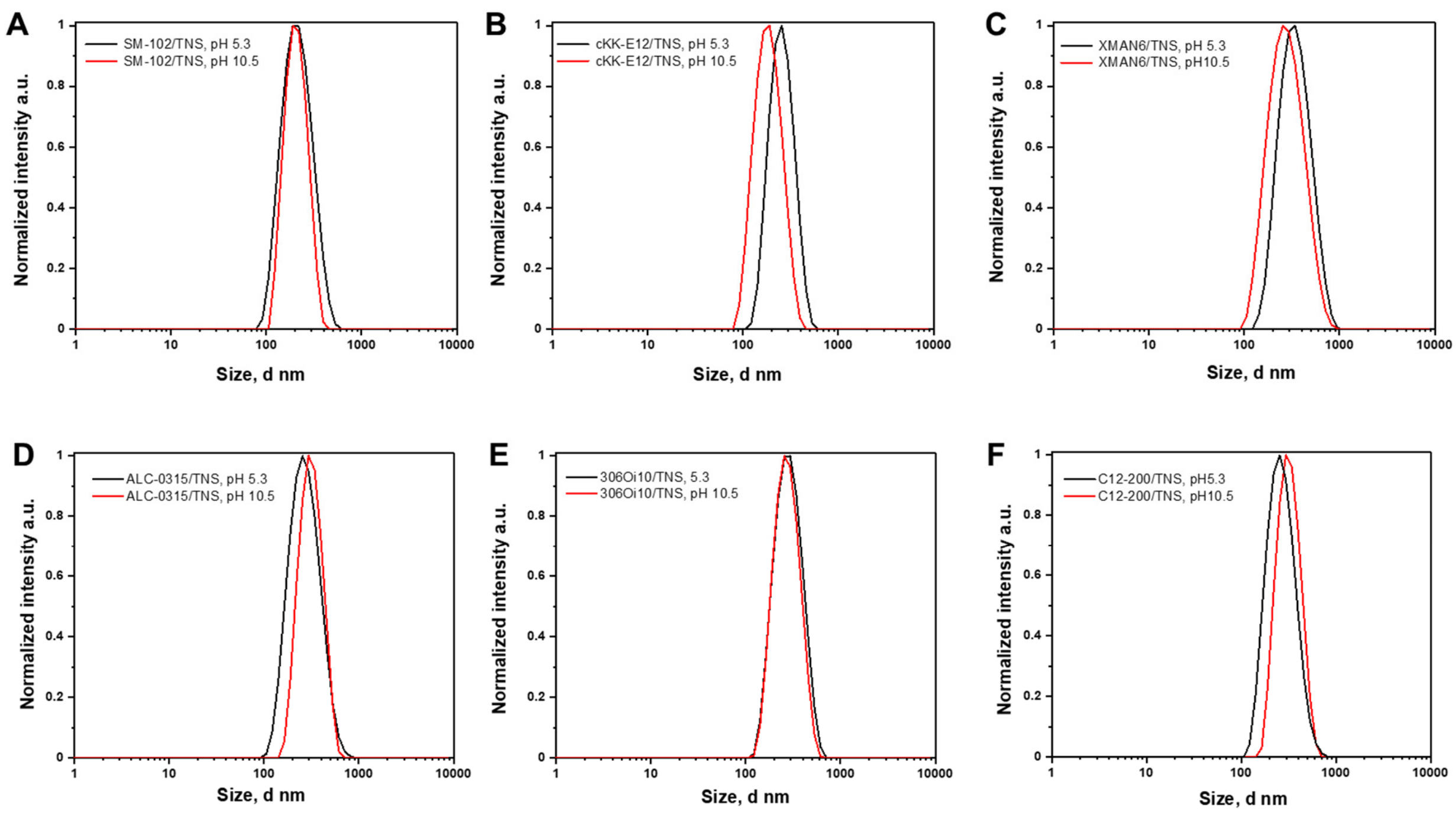

Figure S6. DLS measurements of lipidoid-only assemblies with TNS used for the measurement of pK_a^{app} at pH 5.3 and 10.5. A) SM-102, B) cKK-E12, C) XMAN6, D) ALC-0315, E) $306O_{i10}$, F) C12-200. The particle size distribution is represented as volume-weighted hydrodynamic diameter.

References

[1] Andrew Geall, Vaccine for Eliciting Immune Response Comprising RNA Encoding an Immunogen and Lipid Formulations Comprising Mole Percentage of Lipids, **2023**, US 11638693 B2.
